# Screening failure in systemic sclerosis randomized trials: reporting, rates, causes and trends over time

**DOI:** 10.1093/rap/rkag018

**Published:** 2026-01-31

**Authors:** Delphine Sophie Courvoisier, Barbara Russo, Iulia-Simona Chirică, Michele Iudici

**Affiliations:** Division of Rheumatology, Geneva University Hospitals and University of Geneva, Geneva, Switzerland; Division of Dermatology and Venereology, University Hospitals of Geneva, Geneva, Switzerland; Rheumatology Unit, Ensemble Hospitalier de La Côte, Morges, Switzerland; University of Medicine and Pharmacy “Carol Davila”, Bucharest, Romania; Department of Rheumatology, “Dr. Ion Cantacuzino” Clinical Hospital, Bucharest, Romania; Division of Rheumatology, Geneva University Hospitals and University of Geneva, Geneva, Switzerland

**Keywords:** systemic sclerosis, randomized controlled trials, epidemiology

## Abstract

**Objectives:**

To evaluate the completeness of reporting of pre-randomization patient’ flow in SSc randomized controlled trials (RCTs) and to estimate the extent and reasons for screening failure.

**Methods:**

We searched SSc RCTs indexed in PubMed from 2000 to 2024. We recorded key trial features and checked whether they provided information on patient flow before randomization. We collected information on the adequacy of reporting of the pre-randomization phase, the number of patients screened and the extent and reasons of screening failure. Data were summarized as number (percentage) for qualitative variables and median (interquartile range) for continuous variables.

**Results:**

Of the 127 SSc RCTs retrieved, 52.9% reported patient flow before randomization, 21.2% of those published before 2011 and 65.1% of those published after. The most commonly used terms were ‘screened’ in 33 studies (50%) and ‘assessed for eligibility’ in 29 studies (44%). Of 10 043 patients screened, 5147 (51%) were considered screening failure. The median proportion of screening failures was 36% (IQR 20–58), with higher rates in studies testing non-pharmacologic interventions, lacking industry funding, lacking double-blinding or not including a placebo arm. Reasons for screening failure and their frequency were detailed for 3510 screening failure patients (68.2%). The main reasons were not meeting the eligibility criteria and patient refusal, which accounted for 72.5% and 20.8% cases, respectively. Screening failure remained stable over time.

**Conclusions:**

Reporting of screening procedures in SSc RCTs has improved over time but remains suboptimal. Most screening failures are due to patient ineligibility, followed by patient refusal, which continues to represent a significant barrier to enrolment.

Key messages

- The reporting of screening procedures in SSc RCTs has improved over time but remains suboptimal.
- Most screening failures are due to patient ineligibility, followed by patient refusal, which continues to pose a significant barrier to enrolment.
- Understanding the extent of and underlying reasons for limited patient participation is a crucial step toward broadening trial inclusivity.

## Introduction

SSc is a rare, multisystemic and potentially disabling disease with a pressing need for more effective treatments. Conducting trials in rare diseases poses significant challenges due to the low disease prevalence and the sparse distribution and limited number of expert centres [1, 2]. Approximately one-third of trials recruiting patients with SSc fail, mostly for poor accrual rates [1] and patient enrolment in trials for this disease has been consistently slow in past years [3].

An important aspect of trial conduct is the initial screening process, during which a given patient is considered for eligibility [4]. During the screening process, consent is obtained from patients who are then clinically evaluated and undergo trial-specific laboratory tests and imaging studies. This step could take some days and needs financial resources (estimated up to US$3000 per patient) [5], time and healthcare professional engagement. Understanding the rate and reasons for screening failures can help identify and potentially modify the main criteria limiting patient inclusion, offers clues about patient acceptance of the treatment under investigation, and evaluates the alignment of the study protocol with patient needs [6]. Additionally, the analysis of screening failures provides key insights into whether the study population is representative of patients seen in daily practice. Reporting the number of patients who fail screening and providing detailed reasons for their exclusion is recommended by the Consolidated Standards of Reporting Trials 2010 guidelines [7] and from the Good Clinical Practice statement [8].

Currently there are no published data on the rates or reasons for screening failures in SSc randomized controlled trials (RCTs). It remains unclear whether the primary barriers preventing potentially eligible patients from participating in clinical trials stem from unmet eligibility criteria, patient refusal or other factors. Addressing this gap in knowledge is essential to developing strategies that reduce the efforts and costs associated with screening failures and ultimately facilitate greater participation of SSc patients in clinical research. We conducted an analysis of SSc RCTs published since 2000 to evaluate the completeness of reporting of pre-randomization patient flow and to estimate the extent and reasons for screening failure.

## Methods

We followed the Preferred Reporting Items for Systematic Reviews and Meta-Analyses (PRISMA) statement, with the exception of those relevant only to meta-analyses (e.g. risk of bias assessment) [9]. We searched MEDLINE (via PubMed) on 31 May 2024 for RCTs on SSc published from 2000 onward. The search combined free-text and MeSH terms for SSc and incorporated the Cochrane Highly Sensitive Search Strategy filter for RCTs. Full search strings and parameters are available in Supplementary Data S1 and described elsewhere [3].

### Eligibility criteria

#### Inclusion criteria

We included primary reports of SSc RCTs published since 2000. We defined an RCT as a clinical study randomly allocating participants to different interventions. We identified studies according to whether they reported information about the screening phase. The main analysis was conducted on the sample of studies where this information was available.

#### Exclusion criteria

Studies including patients with scleroderma-like disorders such as morphoea, localized scleroderma or other scleroderma-like diseases (graft-versus-host disease, toxic-related, etc); secondary publications of RCTs (open-label extension, post hoc analysis); non-randomized studies; observational studies; meeting abstracts and studies not in the English language or published before 2000. We put no restriction for treatment, outcome or study phase. Data collection is detailed in Supplementary Data S1 and aligns with that described in a previous publication [3].

### Data extraction and management

Two authors (I.S.C. and B.R.) extracted the data using a standardized form and a third author (M.I.) checked them for consistency. Consensus was reached by discussion. A comprehensive description of the data items collected from each included study (e.g. study characteristics, interventions, patient subsets and complications assessed) is available in the Supplementary Data S1. We assessed how studies documented the flow of patients prior to randomization, including the terminology used to describe this group (e.g. ‘screened patients’, ‘patients considered for eligibility’, etc.). For studies reporting this information, we documented whether the authors specified the number of patients who failed screening as well as the reasons for these failures. If a single reason for screening failure was reported for all screened patients (e.g. ineligibility), we assumed there were no additional reasons (e.g. no patient refusals). However, if reasons for screening failure were provided for only a subset of patients, we considered other reasons as unknown.

The primary outcome was the proportion of screening failures, referring to individuals who underwent screening but were not enrolled in the trial. Our definition incorporates the screening phase (the identification of potentially eligible participants and entry onto a screening or recruitment log) and eligibility assessment (checks against essential inclusion/exclusion criteria in the trial protocol to establish suitability for the trial) [6]. Details on how we defined the funding source, identified the recruiting countries, determined the number of sites and categorized primary outcomes as either ‘patient important’ or ‘surrogate’ are provided in the Supplementary Data S1 and in a separate study we conducted on this topic [3].

### Data analysis

Data were summarized as number (percentage) for qualitative variables and median [interquartile range (IQR)] for continuous variables. Continuous variables were compared with Student’s *t*-test, Wilcoxon test or Kruskal-Wallis test and categorical variables with the chi-squared test or Fisher’s exact test, as appropriate. The screening failure rate for each trial was calculated as the percentage of patients who failed the screening out of the total number of patients screened. This outcome variable was non-normally distributed and all analyses were done using the Wilcoxon rank-sum test. The trial-level factors previously known to impact screening failure were strongly collinear, precluding multivariable analyses.

Data analysis was performed using R version 4.4.2 (R Foundation for Statistical Computing, Vienna, Austria). *P*-values ≤ 0.05 were considered significant. Ethical approval was not required (study not involving human participants).

## Results

### Features of trials reporting *vs* those not reporting information on screening period

**Figure 1:**
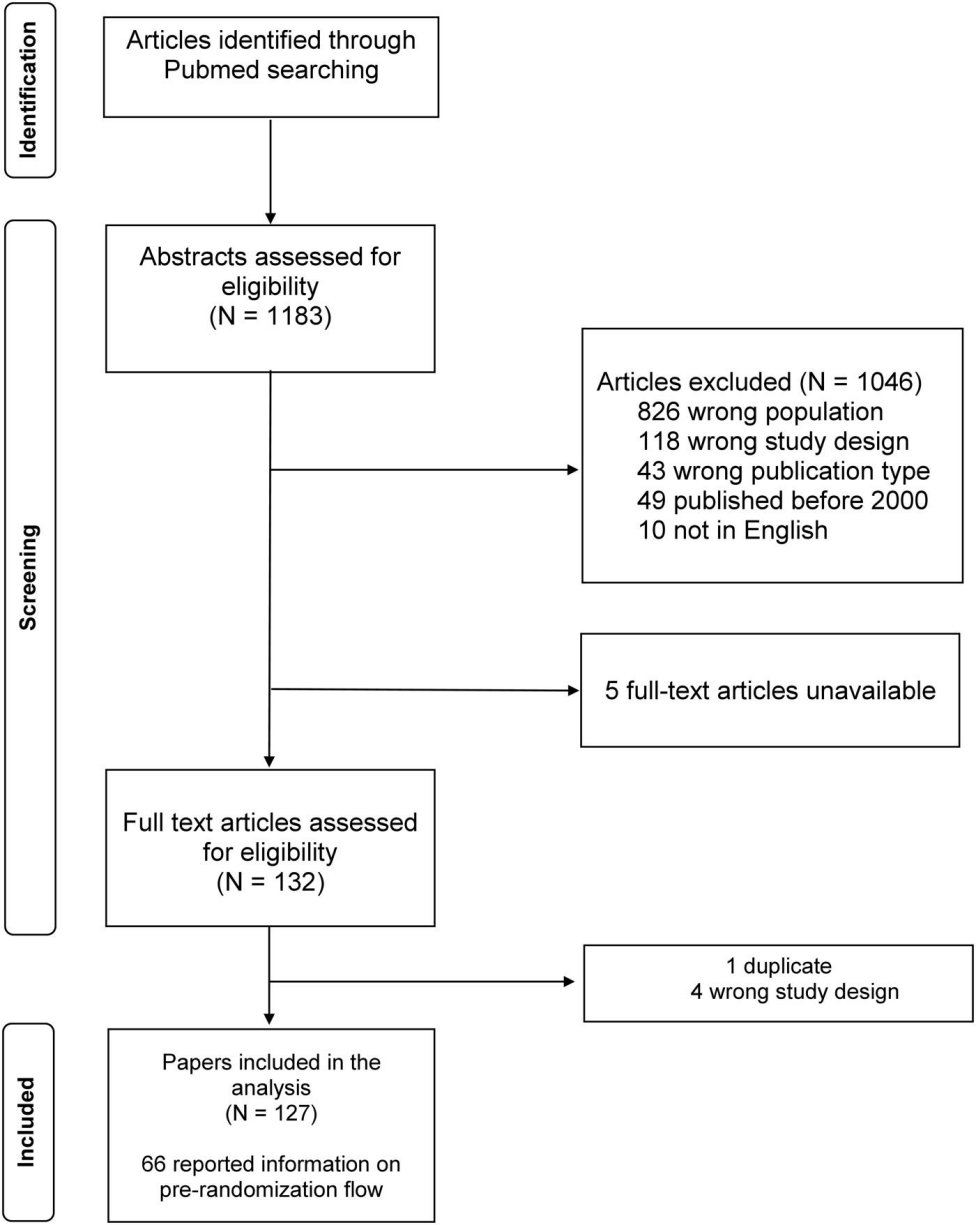
Flow chart of search strategy

Among the 127 RCTs identified (flow chart shown in Fig. 1), 66 articles (52.0%) reported information on patient flow in the pre-randomization phase and were included in the main analysis. Three studies that reported only the number of eligible patients (*n* = 2) or the number of patients who consented to participate (*n* = 1) without providing information on screening were excluded. Among included studies, most started recruiting after 2010 [year of the publication of the CONSORT statement [7]; *n* = 59 (89.4%)], although 33 studies starting recruitment after 2010 still did not report pre-randomization phase patient flow (54.1%), a significant difference in reporting (*P* < 0.001). A total of 45 studies included a CONSORT flow chart detailing patient flow through the study. Included studies were also more likely to test non-pharmacologic treatments (Supplementary Table S1).

The studies providing information on screening/assessment for eligibility (*n* = 66) were mostly single-country [*n* = 48 (72.7%)], equally funded by industry [*n* = 31 (46.9%)] or other funders [*n* = 30 (45.5%)] and were conducted in Europe [*n* = 17 (25.8%)] or North America [*n* = 19 (28.8%)]. The median sample size was 42 patients (IQR 27–84), with 10 RCTs (15.2%) having recruited >100 patients (Table 1).

**Table 1:** Descriptive characteristics and proportion of screening failure.

Characteristics	Studies, n (%)	Screening failure, % (IQR)	P-value
	66 (100)	36 (20–58)	
Funding (not stated: 5)			0.043
Industry	31 (46.9)	29.2 (16.2–29.3)	
Non-industry	30 (45.4)	49.6 (20.8–64.0)	
Location			0.792
Europe	17 (25.7)	38.0 (12.0–66.2)	
North America	19 (28.7)	31.1 (16.7–57.5)	
Central and South America	3 (4.5)	71.7 (19.8–78.6)	
Asia	11 (16.6)	37.3 (17.6–57.6)	
Africa	1 (1.5)	40.0^a^	
Intercontinental	15 (23.0)	33.3 (29.2–61.0)	
Country			0.929
Single country	48 (73.0)	37.6 (18.1–57.7)	
International	18 (27.0)	32.7 (24.0–61.6)	
SSc subset			0.720
dcSSc	19 (28.7)	38.2 (20.8–60.9)	
lcSSc	4 (6.0)	34.9 (6.0–84.0)	
Both	43 (65.3)	33.3 (17.5–48.4)	
Complication studied			0.644
Skin	15 (22.7)	32.2 (17.5–47.9)	
Lung	7 (10.6)	37.3 (29.2–44.7)	
Gastrointestinal	5 (7.6)	29.2 (21.4–48.9)	
Raynaud’s/digital ulcers	15 (22.7)	38.4 (12.3–66.0)	
Hand function/musculoskeletal	10 (15.2)	59.2 (30.0–67.8)	
Other	14 (21.2)	32.6 (24.1–58.6)	
Intervention			
Pharmacologic	33 (50.0)	31.3 (17.5–41.4)	< 0.001
Non-pharmacologic	33 (50.0)	48.4 (21.9–64.1)	
Blinding			0.028
Less than double blind	24 (36.3)	54.2 (29.8–64.2)	
Double blind and over	42 (63.7)	31.6 (17.3–43.3)	
Phase (not stated: 29)			0.437
1/2	28 (42.4)	30.0 (19.0–44.0)	
2/3	9 (13.6)	38.2 (19.8–44.7)	
Randomisation ratio (not stated: 1)			0.214
Equal	57 (86.3)	38.2 (19.9–60.2)	
Skewed	8 (12.1)	28.3 (16.9–37.9)	
Comparator			0.027
Not any placebo arm	26 (39.4)	49.6 (31.2–64.0)	
At least a placebo arm	40 (60.6)	30.1 (16.9–44.2)	
Primary endpoint (not stated: 1)			
Patient important	47 (71.2)	34.6 (17.6–57.6)	0.560
Surrogate	16 (24.2)	38.3 (26.0–59.0)	

a only one study available.

### Screening failure

**Figure 2:**
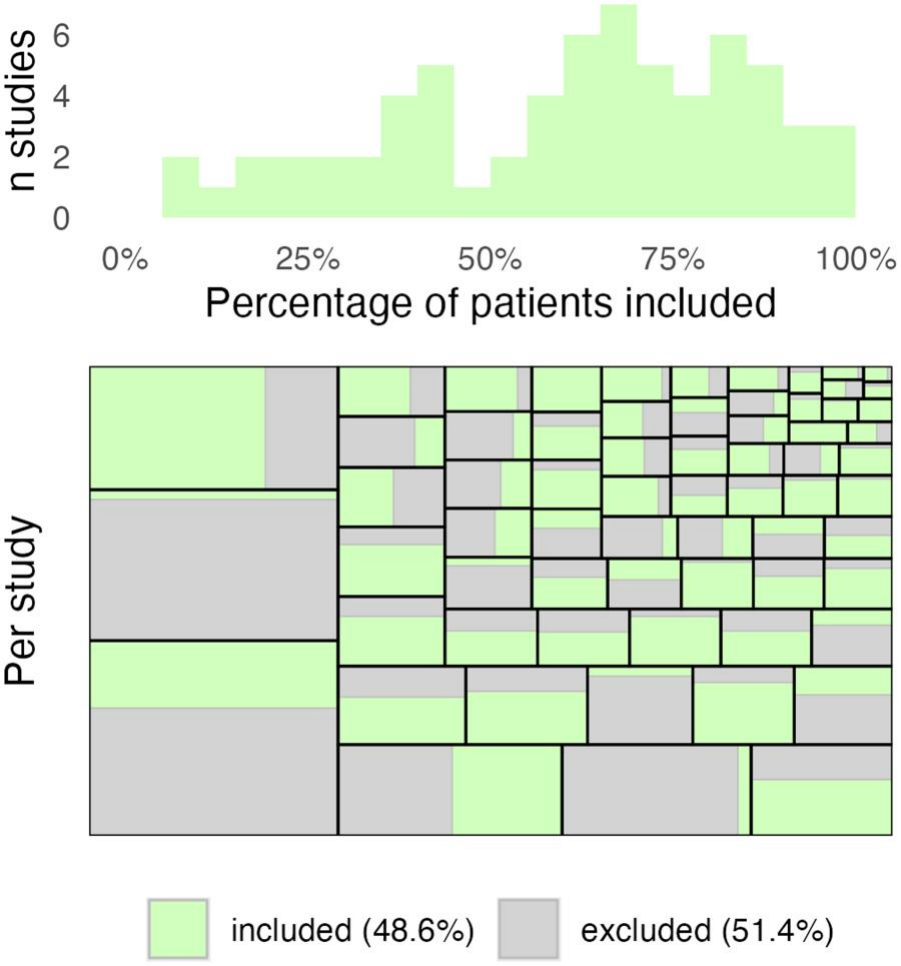
Bar plot of the percentage of screened patients included overall (top panel) and for each study (bottom panel)

The most commonly used terms to describe patient flow in the pre-randomization phase were ‘screened’ in 33 studies (50.0%) and ‘assessed for eligibility’ in 29 studies (43.9%). Of 10 043 patients screened/assessed for eligibility, 5147 (51.2%) were considered a screening failure. The median proportion of screening failures in the overall sample was 36.0% (mean 39.5%), but there was large variability (IQR 19.8–57.7%; minimum 0%, maximum 93.6%) (Fig. 2).

### Reasons for screening failure

Reasons for screening failure and their frequency were reported in 49 of 66 included studies (74.2%) and detailed for 3510 of 5147 patients who experienced screening failure (68.2%). Overall, the main reasons were not meeting the eligibility criteria [*n* = 2545 (72.5%)] and patient refusal [*n* = 733 (20.8%)]. These proportions were reproduced within trials, with a median of 73.9% (IQR 48.6–93.5) of screened patients failing to be randomized due to ineligibility and 18.4% (IQR 5.6–38.3) failing due to refusal.

Only a small number of studies (*n* = 11) provided reasons for patients not meeting eligibility criteria or refusing participation (information available for 551 patients). The primary reasons for failing eligibility criteria were not meeting disease-specific inclusion criteria (*n* = 205), having comorbidities (*n* = 39) or receiving concomitant treatments (*n* = 31). Reasons for patient refusal were reported in only two studies and described vaguely. These included issues such as ‘declined to travel/schedule conflicts’, ‘lack of interest’, ‘feeling overwhelmed by a recent diagnosis’, ‘family obligations’, ‘transportation difficulties’ and ‘limited availability’.

### Factors associated with screening failure

The screening failure percentage was significantly higher in studies examining non-pharmacologic interventions, those without industry funding, those lacking double-blinding and those without a placebo arm (Table 2). All factors were strongly associated with each other, preventing multivariable analyses. The proportion of screening failure remained stable over time, with no association with publication year (Spearman’s ρ = 0.10, *P* = 0.42).

**Table 2:** Median differences in screening failure rates by trial-level characteristics.

Characteristics	Difference in			*P*-value
	Median	Quartile 1	Quartile 3	
Less than double blind (comparator: double blind and over)	22.6	13.7	22.4	<0.001
Patient-important outcome (comparator: surrogate outcome)	−3.7	−9.0	−0.5	0.56
Equal randomization (comparator: skewed randomization)	9.9	2.7	25.6	0.22
Pharmacologic intervention (comparator: non-pharmacologic intervention)	−17.2	−5.5	−24.3	0.050
Industry funding (comparator: non-industry funding)	−20.4	−5.0	−25.2	0.04
No placebo arm (comparator: at least a placebo arm)	19.5	15.0	20.7	0.03

## Discussion

Herein we show that reporting of screening procedures in SSc RCTs has improved after CONSORT statement publication, but it is still suboptimal. Where reported, data point out that on average two SSc patients must be screened to enrol one, with no improvement over time. The highest number of screening failures was observed in non-industry-funded studies and in those testing non-pharmacologic interventions, but this observation can be biased by different reporting practices. Reasons for screening failures were reported in only a minority of SSc RCTs published since 2000 (≈39%) and in 70% of those providing data on pre-randomization patient flow. Among these, patient ineligibility accounted for the majority (≈70%) of screening failures, followed by patient refusal (≈20%). In most cases, authors did not provide sufficient detail to thoroughly analyse the specific reasons for ineligibility or patient refusal.

These findings raise several considerations. First, for most patients who express interest in participation by agreeing to be screened, failing to meet eligibility criteria is the primary barrier. This observation is consistent with findings in other medical fields. For instance, Hasan *et al.* [10] reported that 74% of screening failures in retina trials were due to unmet eligibility criteria. Similarly, Wong *et al*. [11] found that 72% of patients screened for bladder cancer trials were deemed ineligible. While strict eligibility criteria are designed to enhance internal validity, they often do so at the cost of limiting the generalizability of study results [12]. Although such trade-offs can sometimes be justified to ensure patient safety and scientific rigor, eligibility criteria in clinical trials are often overly restrictive. The extent to which this issue affects SSc RCTs remains to be investigated. A few years ago we evaluated what proportion of SSc patients in the European Scleroderma Trials and Research (EUSTAR) cohort would have been eligible for randomized trials conducted over a 5-year period [13]. We found that ineligibility was largely driven by treatment- and safety-related criteria, although we could not determine whether these exclusions were justified [13]. Emerging evidence suggests that even recent clinical trials continue to impose eligibility criteria that are often arbitrarily defined and not clearly linked to known safety concerns of the investigational therapy [14]. Overly restrictive eligibility criteria limit external validity by excluding patients typically seen in clinical practice, making trial results less applicable to real-world SSc populations. This can be improved by adopting broader, evidence-based eligibility criteria, using more pragmatic or adaptive trial designs and aligning study populations with well-characterized real-world cohorts such as those from the EUSTAR database.

Participant refusal emerged as the second most frequent cause of screening failure, although the reasons behind these refusals were rarely reported. Only two studies provided specific reasons for these refusals, underscoring a substantial evidence gap. Understanding why a substantial proportion of eligible patients with SSc decline participation after screening is crucial to improve trial enrolment, one of the key difficulties in clinical research [15]. Contributing factors may include distrust of the healthcare system, negative perceptions of clinical research, disease-specific challenges (e.g. fatigue, anxiety, disability), trial-related burdens (e.g. frequent study visits, lack of travel reimbursement) and site-specific recruitment barriers [16–18]. As patient withdrawal is a potentially preventable cause of screening failure [16], implementing standardized refusal logs and enhancing physician counselling may help identify and address modifiable barriers. Further research is needed to explore these contributing factors in depth. Additionally, actively incorporating patient advisory input early in protocol development is essential to promoting more inclusive, diverse and successful trial participation [19].

We observed a higher rate of screening failure in studies that tested non-pharmacologic interventions, were not industry funded, lacked double-blinding and did not include a placebo arm. Research in other fields shows inconsistent results, with some studies indicating a higher risk of screening failure for surgical interventions [10] or for trials funded by industry [16]. However, these findings should be interpreted with caution, as differences in the extent and quality of reporting across studies may bias the results. For example, reporting may be more rigorous or complete in industry-funded studies, potentially influencing the observed rates of screening failure. Moreover, there is no standardized definition of patient flow across studies. In our sample, roughly half of the studies reported the number of ‘screened patients’, while the other half provided data on ‘patients considered for eligibility’. While screening is formally defined as identifying potentially eligible participants and entering them into a screening log, and eligibility assessment as evaluating inclusion and exclusion criteria [6], the two terms are often used interchangeably. Moreover, CONSORT guidelines do not clearly specify which step requires detailed reporting [7]. This lack of clarity and consistency limits the ability to reliably analyse study-level factors influencing screening failure rates in SSc RCTs. Taken together, these issues underscore the importance of adopting standardized definitions and reporting practices for patient flow in RCTs.

Our analysis was limited to the information reported in published studies, as we did not have access to individual screening logs. Access to these logs could have provided more detailed insights into the causes of screening failure [20]. This study has several strengths, notably the inclusion of research spanning >2 decades, which provides a comprehensive long-term perspective on the topic. Additionally, the scarcity of comparable data in the existing literature highlights the study’s originality and its valuable contribution to filling a significant gap in current knowledge.

In conclusion, information relative to patients screened is lacking in almost half of the trials. Among studies indicating how many patients were screened, half of these patients were not included in RCTs. Understanding the extent and underlying reasons that limit the participation of these patients is a crucial step toward broadening trial inclusivity. It is particularly important to evaluate the impact of poorly justified exclusion criteria on patient recruitment, as well as to explore the factors behind patient’s refusal. Gaining these insights could be instrumental in reducing trial-related costs, minimizing the burden associated with screening failures and ultimately promoting greater participation of SSc patients in clinical research.

## Supplementary Material

rkag018_Supplementary_Data

## Data Availability

The data are available from the corresponding author upon reasonable request.
