# Supplementary material for: Screening failure in systemic sclerosis randomized trials: reporting, rates, causes and trends over time
Source: Rheumatol Adv Pract. 2026 Jan 31;10(2):rkag018. doi: 10.1093/rap/rkag018 (PMC13075944; doi:10.1093/rap/rkag018)
Supplement: rkag018_Supplementary_Data [file rkag018_supplementary_data.docx]

**Supplementary Data S1**

**Search strategy**

We performed an electronic search of MEDLINE via PubMed on 31 May 2024 to identify RCTs on SSc published since 2000. We used the following combination of free terms and MeSH terms (“CREST”[tiab] OR "Scleroderma, Systemic"[Mesh] OR “Systemic sclerosis”[All Fields] OR “scleroderma”[All Fields]) to identify papers on systemic sclerosis. The Cochrane Highly Sensitive Search Strategy was applied for identifying randomized trials (Glanville JM, Lefebvre C, Miles JN, Camosso-Stefinovic J. How to identify randomized controlled trials in MEDLINE: ten years on. J Med Libr Assoc. 2006;94:130-6).

**Data collection**

All retrieved references were downloaded in the free online program Rayyan (Qatar Computing Research Institute, https://www.rayyan.ai), a systematic review web-based application. Two researchers (ISC, BR) independently checked each title and abstract to exclude irrelevant papers. The full text article was retrieved to confirm eligibility if information in the abstract was unclear or insufficient. The same reviewers then independently examined full-text articles to determine eligibility. Consensus was reached by discussion in case of disagreement. A third reviewer (MI) was available in case of unsolved disagreement. We documented the primary reason for exclusion of full-text articles.

**Data extraction and management: number of sites, country, funding, patient-important versus surrogate outcome**

If the number of centers was not clearly specified in the published manuscript or supplementary file, we obtained this information from online trial registration repositories. When authors only mentioned the contributing university, we assumed one site per university.

Information about the enrolling country(ies) was obtained as follows: first, we checked whether the recruitment centers were reported in the full text; if no explicit information was available, we looked at the location of the institutions linked to the authors; if all the institutions were from the same country, we considered the study to have taken place in that country; in case the authors came from different countries and if information about recruiting centers was lacking in the full-text, we checked the supplementary online material, the published protocols, or the trial registration online repositories (e.g. WHO - International Clinical Trials Registry Platform, Clinicaltrials.gov). If the authors were from institutions in different countries/continents, we considered the study to be international/intercontinental.

A study was considered being industry-funded if the sponsor or one of the collaborators was industry. For each RCT, we collected primary outcome(s).

Each primary outcome was independently classified by 2 of the authors (BR and MI) as ‘patient important’ or ‘surrogate’ outcomes according to previous works on this topic (Gandhi GY, Murad MH, Fujiyoshi A, Mullan RJ, Flynn DN, Elamin MB, et al. Patient-important outcomes in registered diabetes trials. Jama. 2008;299:2543-9). We classified patient-important outcomes as measures that directly impact on quality of life, such as major morbid events (e.g., end-stage lung disease, loss of hand function, etc) or minor morbid events (e.g., pain and functional status); surrogate outcomes were classified as measures that may indicate disease progression and increased risk for patient-important outcomes, or as assessed response to physiologic or laboratory testing without direct tangible effects on patients (e.g., capillaroscopic pattern, worsening of a respiratory parameter, etc.). In case of disagreement, consensus was reached by discussion.

**Supplementary Table S1.** Descriptive characteristics of studies reporting or not information on screening phase.

|  | Studies reporting information on screening, n | Studies not reporting information on screening, n | P value |
| --- | --- | --- | --- |
|  | 66 | 61 |  |
| **Date of recruitment start** (not stated: 36) |  |  |  |
| Before 2011 | 14 (21.2) | 17 (27.8) | 0.021 |
| 2011 - 2024 | 43 (65.1) | 17 (27.8) |  |
| **Funding** (not stated: 18) |  |  |  |
| Industry | 31 (46.9) | 27 (44.3) | 0.073 |
| Non-Industry | 30 (45.4) | 21 (34.4) |  |
| **Location** |  |  |  |
| Europe | 17 (25.7) | 31 (50.8) | 0.081 |
| North America | 19 (28.7) | 12 (19.7) |  |
| Central - South America | 3 (4.5) | 2 (3.3) |  |
| Asia | 11 (16.6) | 7 (11.5) |  |
| Africa | 1 (1.5) | 2 (3.3) |  |
| Intercontinental | 15 (23.0) | 7 (11.5) |  |
| **Country** |  |  | 0.132 |
| Single-country | 48 (73.0) | 52 (85.2) |  |
| International | 18 (27.0) | 9 (14.8) |  |
| **SSc subset** (not stated: 5) |  |  | 0.125 |
| dcSSc | 19 (28.7) | 15 (24.6) |  |
| lcSSc | 4 (6.0) | 4 (6.6) |  |
| both | 43 (65.3) | 37 (60.7) |  |
| **Complication studied** |  |  | 0.136 |
| Skin | 15 (22.7) | 16 (26.2) |  |
| Lung | 7 (10.6) | 5 (8.2) |  |
| Gastrointestinal | 5 (7.6) | 6 (9.8) |  |
| Raynaud’s/digital ulcers | 15 (22.7) | 20 (32.8) |  |
| Hand function / Musculoskeletal | 10 (15.2) | 1 (1.6) |  |
| Other | 14 (21.2) | 13 (21.3) |  |
| **Intervention** |  |  | 0.018 |
| Pharmacologic | 33 (50.0) | 44 (72.1) |  |
| Non-pharmacologic | 33 (50.0) | 17 (27.9) |  |
| **Blinding** |  |  |  |
| Less than double blind | 24 (36.3) | 28 (45.9) | 0.362 |
| Double blind and over | 42 (63.7) | 33 (54.1) |  |
| **Randomisation ratio**  (Not stated: 1) |  |  | 0.291 |
| Equal | 57 (86.3) | 49 (78.9) |  |
| Skewed | 8 (12.1) | 12 (21.1) |  |
| **Comparator** |  |  |  |
| Not any placebo arm | 26 (39.4) | 24 (39.3) |  |
| At least a placebo arm | 40 (60.6) | 37 (60.7) | 1.00 |
| **Primary endpoint**  (Not stated: 1) |  |  |  |
| Patient-important | 47 (71.2) | 41 (66.7) | 0.442 |
| Surrogate | 16 (24.2) | 20 (33.3) |  |

**dcSSc.** diffuse cutaneous systemic sclerosis; **lcSSc.** limited cutaneous systemic sclerosis.
